# Ca^2+^ and cAMP open differentially dilating synaptic fusion pores

**DOI:** 10.1242/jcs.261026

**Published:** 2023-07-04

**Authors:** Dinara Bulgari, Samantha L. Cavolo, Brigitte F. Schmidt, Katherine Buchan, Marcel P. Bruchez, David L. Deitcher, Edwin S. Levitan

**Affiliations:** ^1^ Department of Pharmacology and Chemical Biology, University of Pittsburgh, Pittsburgh, PA 15261, USA; ^2^ Department of Chemistry, Carnegie Mellon University, Pittsburgh, PA 15213, USA; ^3^ Department of Chemistry, Carnegie Mellon University, Pittsburgh, PA 15213, USA; ^4^ Department of Biology, Carnegie Mellon University, Pittsburgh, PA 15213, USA; ^5^ Molecular Biosensor and Imaging Center, Carnegie Mellon University, Pittsburgh, PA 15213, USA; ^6^ Department of Neurobiology and Behavior, Cornell University, Ithaca, NY 14853, USA

**Keywords:** Fusion pore expansion, Neuromuscular junction, Secretory granule, Synaptic transmission, *Drosophila*

## Abstract

Neuronal dense-core vesicles (DCVs) contain neuropeptides and much larger proteins that affect synaptic growth and plasticity. Rather than using full collapse exocytosis that commonly mediates peptide hormone release by endocrine cells, DCVs at the *Drosophila* neuromuscular junction release their contents via fusion pores formed by kiss-and-run exocytosis. Here, we used fluorogen-activating protein (FAP) imaging to reveal the permeability range of synaptic DCV fusion pores and then show that this constraint is circumvented by cAMP-induced extra fusions with dilating pores that result in DCV emptying. These Ca^2+^-independent full fusions require PKA-R2, a PKA phosphorylation site on Complexin and the acute presynaptic function of Rugose, the homolog of mammalian neurobeachin, a PKA-R2 anchor implicated in learning and autism. Therefore, localized Ca^2+^-independent cAMP signaling opens dilating fusion pores to release large cargoes that cannot pass through the narrower fusion pores that mediate spontaneous and activity-dependent neuropeptide release. These results imply that the fusion pore is a variable filter that differentially sets the composition of proteins released at the synapse by independent exocytosis triggers responsible for routine peptidergic transmission (Ca^2+^) and synaptic development (cAMP).

## Teaser

**Summary:** Independent triggers of release (Ca^2+^ and cAMP) induce different levels of fusion pore dilation to vary the composition of proteins released from synaptic dense-core vesicles.

## INTRODUCTION

Neuronal dense-core vesicles (DCVs) contain a wide variety of secretory protein cargoes, including neuropeptides (with molecular masses of 0.6–30 kDa in *Drosophila melanogaster*) and proteases that function in synaptic plasticity and growth (e.g. the 70 kDa tissue plasminogen activator) (Huang et al., 1996; Baranes et al., 1998). Early studies of peptide hormone release from endocrine cells emphasized Ca^2+^-induced DCV emptying by full collapse exocytosis or full fusion that follows fusion pore dilation. However, studies of spontaneous and activity-evoked release by DCVs in the intact *Drosophila* neuromuscular junction (NMJ) with two different imaging approaches have failed to detect DCV emptying and instead produced data consistent with neuropeptide release via fusion pores formed by kiss-and-run exocytosis (Wong et al., 2015; Bulgari et al., 2019). Partial release by kiss-and-run exocytosis of presynaptic DCVs is conducive with the cell biology of neurons: because DCVs are replaced by axonal transport that can take days, kiss-and-run exocytosis prevents rapid depletion of stores at distal sites of release that are slow to refill. However, it is not known whether presynaptic fusion pores allow for release of large DCV cargoes.

Imaging permeation through DCV fusion pores is difficult in native synapses that contain DCVs and small synaptic vesicles (SSVs) because fluid-phase fluorophores used with endocrine cells (e.g. Takahashi et al., 2002) cannot distinguish between the two vesicle types and cutting-edge superresolution microscopy methods that resolve fusion pore dilation rely on *in vitro* preparations (e.g. Anantharam et al., 2011). Likewise, electrical and electrochemical fusion pore measurements (Sharma and Lindau, 2018) are not well suited to typical native co-transmitting synaptic boutons. However, synaptic DCV fusion pores were detected recently using membrane-impermeant malachite green (MG)-based fluorogens and a fluorogen-activating protein (FAP) targeted to the DCV lumen so that, upon opening of a fusion pore, fluorogens pass through the fusion pore into the DCV to bind the FAP with picomolar affinity and generate far-red fluorescence (Bulgari et al., 2019). Because previously used fluorogens varied in shape and constituent moieties, and their hydrodynamic sizes were not characterized (Bulgari et al., 2019), the permeability of presynaptic fusion pores was not studied systematically. Nevertheless, because the fusion pore is a channel and passage of large native DCV cargoes through the fusion pore out of the DCV cannot be imaged without introducing large tags that would alter the results, we reasoned that a series of fluorogens and FAP imaging could methodically probe the permeability of the synaptic DCV fusion pore.

Here, a series of single-chain PEGylated membrane impermeant MG fluorogens and a FAP inserted into the neuropeptide *Drosophila* insulin-like peptide 2 (Ilp2, fusion is denoted Dilp2–FAP, Bulgari et al., 2019) are used at the *Drosophila* NMJ to show that synaptic fusion pores that open spontaneously and in response to activity are not permeable to large DCV cargoes. Then the conundrum of how such proteins can be efficiently released from presynaptic DCVs is resolved by establishing that anchored, activated protein kinase A (PKA) and Complexin open dilating fusion pores to empty DCVs. Thus, different exocytosis triggers produce differential dilation of fusion pores, which, in turn, alters the composition of proteins released at the synapse.

## RESULTS

### Synaptic DCV fusion pore permeability

Fluorogens were synthesized via conjugating single-chain polyethylene glycol (PEG) adducts to MG. Their hydrodynamic sizes were then determined in comparison to proteins by size exclusion fast protein liquid chromatography (FPLC) (see Materials and Methods). The hydrodynamic properties of these fluorogens scaled with molecular mass differently from proteins; for example, the MG conjugate to a 1 kDa PEG chain with a molecular mass of 1.55 kDa behaved in solution like a 4.3 kDa protein. Because the hydrodynamic size of fluorogens was not taken into account previously (Bulgari et al., 2019), here sizes are expressed in terms of apparent protein molecular mass. Specifically, a series of unbranched MG-PEGs with apparent protein molecular masses ranging from 4.3 to 71.1 kDa were examined for activity-evoked FAP responses. As demonstrated with other membrane impermeant fluorogens, such as MG–BTau, FAP responses reflect the opening of fusion pores and the retention of fluorescent MG–Dilp2–FAP complexes that do not exit through fusion pores (Bulgari et al., 2019). However, because optical resolution is not as favorable in the brain for studying release by the soma (Klose et al., 2021), we have no single DCV data from that neuronal compartment. Also, we have not noticed FAP events between boutons and have no data from motor nerves, where the ‘blood–brain barrier’ might prevent access of fluorogens to the axons in motor nerves. Therefore, we studied responses at *Drosophila* muscle 6 and 7 NMJ type Ib boutons.

**Fig. 1.**
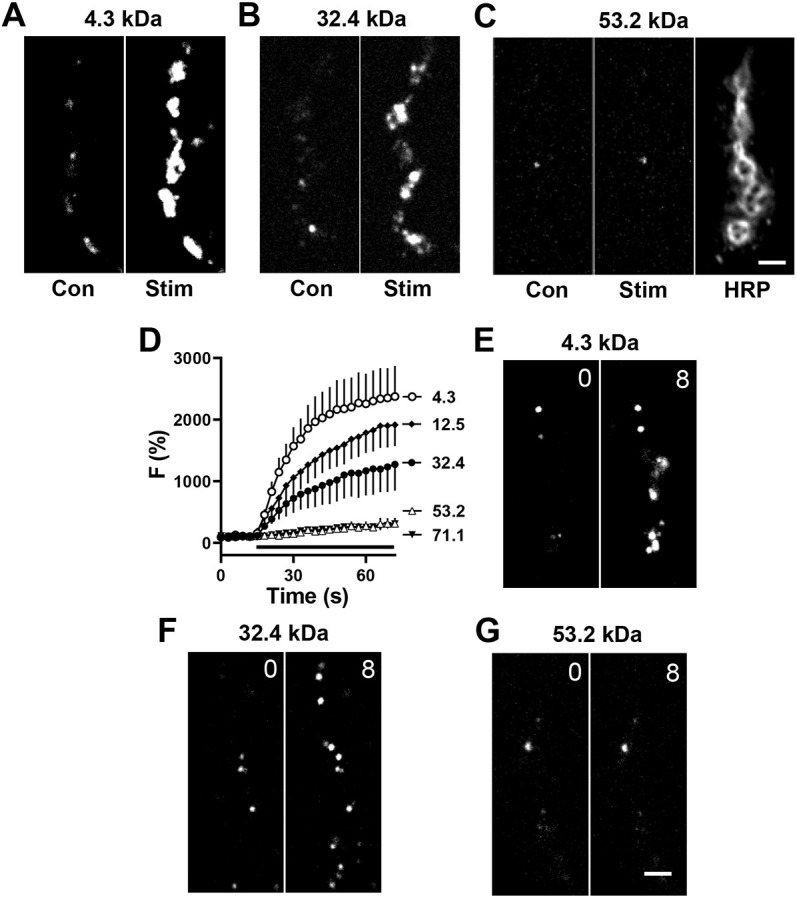
**Permeability of PEG derivatives through DCV fusion pores.** (A–C) Contrast-enhanced fluorescent images of type Ib boutons from Ok6-GAL4 UAS-Dilp2FAP larvae before (Con) and after 70 Hz stimulation (Stim) for 60 s in the presence of 4.3 kDa (A), 32.4 kDa (B) and 53.2 kDa (C) MG-PEG dyes, each at 1 µM. Anti-horseradish peroxidase immunofluorescence (HRP) is shown to indicate location of boutons. (D) Time-course of activity-evoked FAP responses in the presence of MG-PEG dyes normalized to initial labeling. Apparent protein sizes shown in symbol labels. Black bar, 70 Hz stimulation. 4.3 kDa: *n*=8 NMJs (one bouton each, four animals); 12.5 kDa *n*=7 NMJs (one bouton each, four animals); 32.4 kDa : *n*=5 NMJs (one bouton each, five animals); 53.2 kDa *n*=6 NMJs (one bouton each, five animals); 71.1 kDa *n*=6 NMJs (one bouton each, five animals). (E–G) Contrast-enhanced fluorescent images of Dilp2-FAP expressing boutons in the absence of Ca^2+^ after application of 4.3 kDa (E), 32.4 (F) and 53.2 kDa (G) MG-PEGs. Numbers on the images indicate time in minutes. Images in E–G representative of 10–12 experiments. Scale bars: 2 µm.

Responses to 1 min of 70 Hz stimulation led to robust labeling with the 4.3 kDa fluorogen, but were attenuated with larger fluorogens (Fig. 1A–D). For the three smaller fluorogens (4.3, 12.5 and 32.4 kDa), the incremental changes could reflect the slower diffusion of larger fluorogens, the effect of approaching the size cutoff of the fusion pore or be a consequence of complex fusion pore kinetics (Stenovec et al., 2004). However, the two largest fluorogens (53.2 and 71.1 kDa), with incrementally modest increases in hydrodynamic size (because radius is proportional to the cube root of protein molecular mass), barely produced any signals (Fig. 1C,D), thereby indicating poor permeation through synaptic DCV fusion pores that open in response to Ca^2+^ elevation induced by activity. With spontaneous fusion pore openings (Bulgari et al., 2019), the same properties are evident. First, application of fluorogens in the absence of Ca^2+^ produced some immediate labeling of puncta, likely reflecting past release (Bulgari et al., 2019), thus showing that even the large fluorogens reach the bouton surface. However, in contrast with the 4.3 and 32.4 kDa fluorogens, time-dependent labeling was not seen with incubation of the NMJ with the 53.2 kDa fluorogen in the absence of Ca^2+^ for 8 min (Fig. 1E–G). Thus, synaptic fusion pore permeability is sufficient for the release of all *Drosophila* neuropeptides, but excludes DCV cargoes that exceed the fusion pore cutoff, which is above 32.4 kDa.

### Frequency of DCV emptying events

**Fig. 2.**
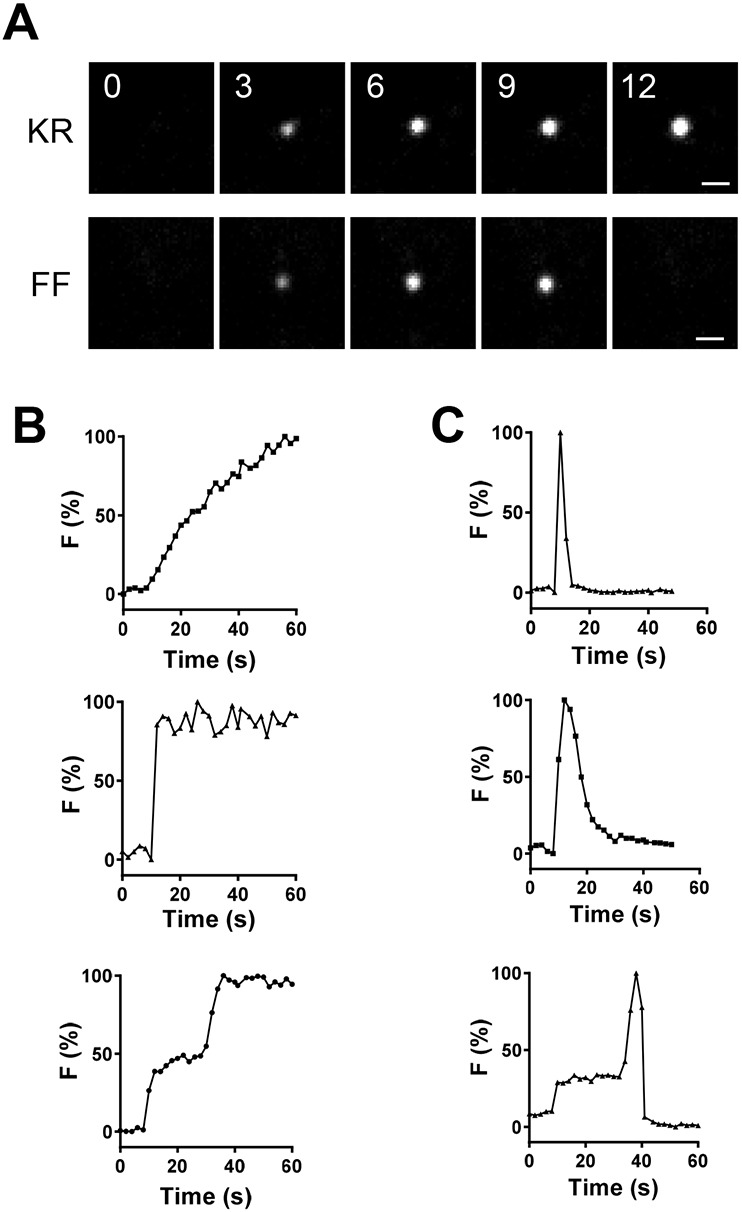
**Individual spontaneous kiss-and-run and full fusion events.** (A) Contrast-enhanced fluorescent images of single labeled Dilp2–FAP presynaptic sites in the absence of Ca^2+^ after application of MG–BTau fluorogen in OK6>Dilp2-FAP type Ib boutons. Upper panels labeled KR show consecutive images of kiss-and-run pore opening, whreas the lower panels labeled FF show consecutive images of full fusions. Numbers on images indicate times in seconds. Scale bar: 1 µm. (B,C) Normalized time courses of representative individual (B) kiss-and-run and (C) full fusion sites. Data representative of ten experiments.

**Fig. 3.**
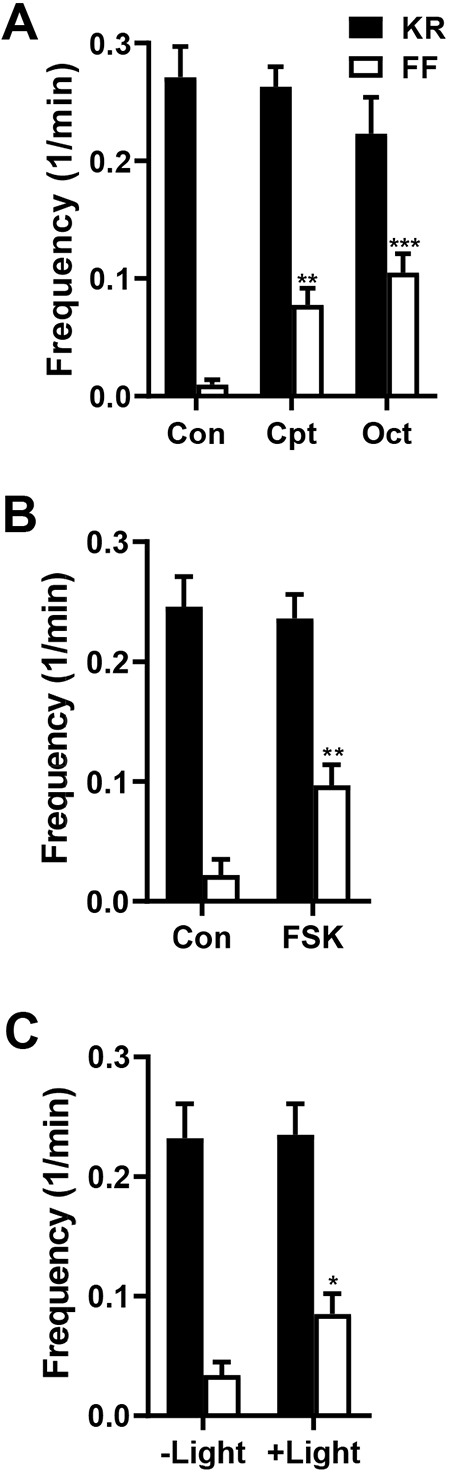
**cAMP increases the frequency of spontaneous full fusions.** (A) Mean±s.e.m. frequency of kiss-and-run (KR, black bars) and full fusions (FF, white bars) in control boutons (Con, *n*=8 NMJs, 71 boutons, five animals), boutons treated with the cAMP agonist 8-parachlorophenyl-thio-cAMP (Cpt, *n*=7 NMJs, 61 boutons, four animals) or boutons treated with octopamine (Oct, *n*=11 NMJs, 141 boutons, five animals) in the presence of MG–BTau. Statistical comparisons are always between like-colored bars; **P<0.01, ***P<0.001 (one-way ANOVA followed by Dunnett's post test). (B) Mean±s.e.m. frequency of kiss-and-run (black bars) and full fusions (white bars) per bouton in DMSO control (Con, *n*=12 NMJs, 91 boutons, six animals) and FSK-treated neurons (FSK, *n*=17 NMJs 144 boutons, ten animals) in the presence of MG–BTau, **P<0.01, unpaired two-tailed *t*-test. (C) Frequency of kiss-and-run (black bars) and full fusions (white bars) in PACα-expressing boutons without illumination (–Light, *n*=11 NMJs, 92 boutons, four animals) and light-activated PACα (+Light, *n*=16 NMJs, 167 boutons, eight animals) in the presence of MG–BTau. Statistical comparisons are always between like-colored bars; *P<0.05, unpaired two-tailed *t*-test. There were no significant differences between kiss-and-run fusion frequency (black bars) in the presented experiments, unpaired two-tailed *t*-test. All data derived from Ib boutons of OK6>Dilp2–FAP animals. 1/min, min^−1^.

Given the above synaptic DCV fusion pore permeability, we explored whether NMJ DCVs ever empty to efficiently release large cargoes, a process often referred to as full fusion (FF). For this purpose, 1 µM MG–BTau (a very water soluble, relatively small fluorogen) was used to detect spontaneous events that occur in the absence of extracellular Ca^2+^ (Bulgari et al., 2019), thus enabling imaging of individual release sites without interference from surrounding events that are stimulated by activity. Single labeling events via kiss-and-run fusion pores (Fig. 2A, KR) were readily detected by their sustained labeling and occurred with variable kinetics (Fig. 2B, top and middle); overall, they grew over 23.7±2.7 s (mean±s.e.m.; *n*=37) and could persist for the duration of imaging (up to 4 min). However, on rare occasions labeling via the opening of a fusion pore was followed within seconds by the rapid loss of fluorescence, indicative of DCV emptying (Fig. 2A, FF). Again, time courses were variable (Fig. 2C); the lifetime of these events was 17.25±3.81 s with a rise time to peak fluorescence of 5.62±0.53 s (*n*=8). Based on the imaging field of view and the depth of field, such events could not be attributed to undocking and transport of DCVs. Likewise, pH is not a factor, because *Drosophila* DCVs are not very acidic (Sturman et al., 2006) and FAP–MG fluorescence is hardly affected between pH 5 and 8 (Perkins et al., 2018). Rather, following MG fluorogen influx through the fusion pore and binding to the FAP, MG–FAP complexes that normally are retained (i.e. cannot exit through fusion pores) must have been released by fusion pore dilation that resulted in DCV emptying. Interestingly, such full fusions could occur with a DCV that had already had a kiss-and-run event (Fig. 2C, bottom). Overall, DCV full fusions only occurred in 3.6% of exocytotic events at the synapse in these experiments (Fig. 3A, Con; black bar shows kiss-and-run, white bar shows full fusion; frequencies expressed per bouton).

### cAMP evokes opening of dilating fusion pores

Because it seems unlikely that such rare full fusions wholly account for release of large DCV cargo proteins, we considered that signaling might trigger synaptic DCV full fusions. A candidate for such a trigger is cAMP. In endocrine cells (e.g. pituitary lactotrophs, pancreatic β-cells and adrenal chromaffin cells) cAMP effects have been studied in the presence of Ca^2+^, leading to insights into regulation of Ca^2+^-evoked fusion pore behavior, electrical activity and peptide hormone release (Calejo et al., 2013; 2014; Kucka et al., 2013; Guček et al., 2019; González-Santana et al., 2021). However, at the *Drosophila* NMJ, cAMP evokes release from DCVs in the absence of extracellular Ca^2+^ and the presence of a Ca^2+^ chelator without altering cytoplasmic [Ca^2+^] (Shakiryanova et al., 2011; Bulgari et al., 2018). Therefore, we tested whether cAMP affects the frequency of Ca^2+^-independent kiss-and-run and/or full fusion exocytosis events at the NMJ by FAP imaging with MG–BTau. Very large fluorogens were not used in these experiments because their inability to reach the DCV lumen and stabilize FAP dimers that are required for fluorescence (Bulgari et al., 2019) limits detection of both kiss-and-run exocytosis and full fusions; without fusion pore permeation, labeling inside the DCV never occurs and FAP dilution following pore dilation allows low affinity dimers to dissociate and/or diffuse away from the release site.

Four independent experimental approaches showed that cAMP selectively evokes presynaptic DCV full fusions. First, treatment with the membrane-permeant cAMP analog 8-parachlorophenyl-thio-cAMP (CPT, 1 mM) increased the frequency of full fusions without affecting kiss-and-run events (Fig. 3A). Second, bath application of 100 µM octopamine, an insect neuromodulator that elevates bouton cAMP at the NMJ (Maiellaro et al., 2016) and acts via cAMP to promote the growth of NMJ arbors (Koon et al., 2011), also increased the frequency of DCV emptying with no effect on the kiss-and-run event frequency (Fig. 3A, Oct). Third, bath application of 100 µM forskolin (FSK), an adenylate cyclase activator that elevates cAMP in *Drosophila* neurons (Shafer et al., 2008), increased the full fusion frequency without altering kiss-and-run fusion frequency (Fig. 3B). Furthermore, the rise times of responses [25.5±3 s (*n*=23) for kiss and run; 6.0±1.0 (*n*=11) for full fusions; mean±s.e.m.] were unchanged. Although these kinetics might have been affected by efflux of luminal cargoes, the lack of change with cAMP implies that the frequency of one subtype of release is increased without changing the kinetics of individual events. Finally, following motor neuron-specific expression of the photoactivatable adenylate cyclase PACα (Schröder-Lang et al., 2007), blue light activation of the adenylate cyclase increased the frequency of DCV full fusions, again with no change in kiss-and-run frequency (Fig. 3C). Together, these results show that cAMP evokes release from presynaptic DCVs by selectively increasing the frequency of full fusions without affecting ongoing kiss-and-run exocytosis.

### Anchored PKA-R2 is required for cAMP-evoked full fusions

**Fig. 4.**
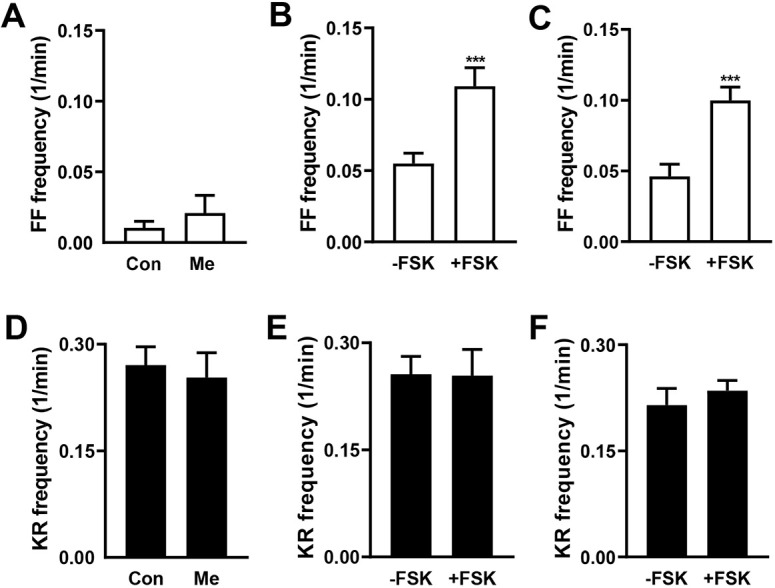
**Epac is not involved in cAMP-induced full fusions.** (A) Frequency of full fusions (FF) in control OK6>Dilp2–FAP boutons (Con, *n*=8 NMJs, 63 boutons, four animals) and pretreated with Epac activator 8-(4-methoxyphenylthio)-2′-O-methyl-cAMP (Me, *n*=8 NMJs, 58 boutons, four animals). No significant difference was detected with unpaired two-tailed *t*-test. (B) Frequency of full fusions in NMJs treated with Epac inhibitor ESI-09 without FSK (−FSK, *n*=10 NMJs, 112 boutons, five animals) and with FSK (+FSK, *n*=7 NMJs, 55 boutons, four animals). ***P<0.001 (unpaired two-tailed *t*-test). (C) Frequency of full fusions in Epac-null flies without FSK (−FSK, *n*=11; 127 boutons, six animals) and with FSK (+FSK) *n*=17 NMJs, (193 boutons, nine animals), ***P<0.001 (unpaired two-tailed *t*-test). (D–F) Frequency of kiss-and-run fusions with conditions corresponding to A–C. Note that there were no significant differences in kiss-and-run fusions for the experimental conditions presented here. 1/min, min^−1^.

The effects of cAMP are mediated by two ubiquitously expressed intracellular cAMP effectors, PKA and the exchange protein directly activated by cAMP (Epac) (de Rooij, 1998). To test for a role of Epac, we began with bath application of 8-(4-methoxyphenylthio)-2′-O-methyl-cAMP (Me, 200 µM), an Epac activator that has been used at the *Drosophila* NMJ (Shakiryanova et al., 2011). However, no changes in the frequencies of emptying or kiss-and-run events were produced (Fig. 4A,D). Furthermore, pretreatment of boutons with the Epac inhibitor ESI-09 (10 µM), which has been used to study release from mammalian DCVs (Guček et al., 2019), did not disrupt the forskolin-evoked increase in full fusion frequency (Fig. 4B) or alter kiss-and-run events (Fig. 4E). Finally, in Epac-null animals (Shakiryanova et al., 2011), the selective effect of forskolin on emptying persisted (Fig. 4C,F). Interestingly, the latter two approaches to reducing Epac function tended to increase basal emptying frequency without eliminating the forskolin effect. This suggests that Epac constrains fusion pore dilation in the synaptic terminal independently of cAMP. But more important here, Epac is not required for cAMP-induced DCV full fusions.

**Fig. 5.**
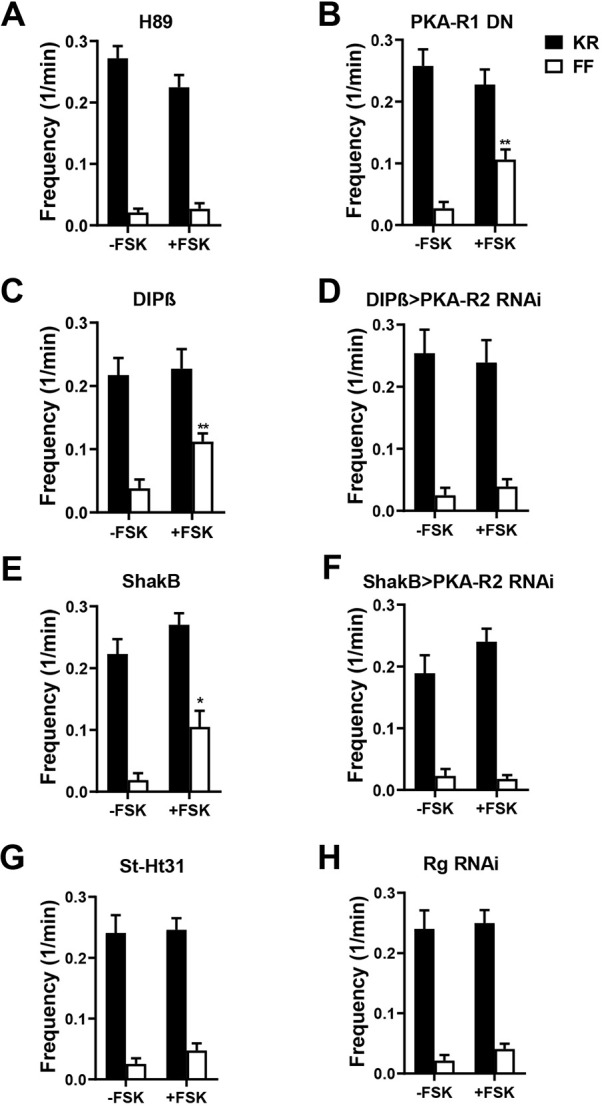
**cAMP-induced full fusions require anchored PKA-R2.** (A–H) Frequency of kiss-and-run (KR, black columns) and full fusions (FF, white columns). (A) Frequency of fusions in OK6>Dilp2–FAP boutons treated with H89 without forskolin (−FSK, *n*=14 NMJs, 132 boutons, six animals) and with forskolin application (+FSK, *n*=9 NMJs, 100 boutons, five animals). (B) Frequency of fusions in OK6>Dilp2–FAP boutons co-expressing dominant-negative PKA-R1 (PKA-R1 DN) with and without forskolin application (−FSK: *n*=8 NMJs, 74 boutons, five animals; +FSK: *n*=14 NMJs, 98 boutons, eight animals). **P<0.01 (unpaired two-tailed *t*-test). (C) Frequency of fusions in DIPβ>Dilp2–FAP boutons with and without forskolin application (−FSK: *n*=8 NMJs, 62 boutons, four animals; +FSK: *n*=9 NMJs, 65 boutons, five animals. **P<0.01 (unpaired two-tailed *t*-test). (D) Frequency of fusions in DIPβ>Dilp2–FAP boutons also expressing PKA-R2 RNAi with and without forskolin application (−FSK: *n*=7 NMJs, 50 boutons, four animals; +FSK, *n*=7 NMJs, 51 boutons, four animals). (E) Frequency of fusions ShakB>Dilp2–FAP boutons co-expressing the Valium20 RNAi control with and without FSK treatment (ShakB) (−FSK, *n*=8 NMJs, 59 Is boutons, four animals; +FSK: *n*=10 NMJs, 67 boutons, five animals). (F) Frequency of fusions in ShakB>Dilp2–FAP boutons co-expressing PKA-R2 RNAi (ShakB>R2 RNAi) with and without FSK treatment (−FSK: *n*=8, 88 Is boutons, four animals; +FSK: *n*=19 NMJs, 166 boutons, eight animals). (G) Frequency of fusions in St-Ht31 pretreated OK6>Dilp2–FAP boutons with and without FSK treatment (−FSK: *n*=12 NMJs, 151 boutons, five animals; +FSK: *n*=20 NMJs, 228 boutons, seven animals). (H) Frequency of fusions in OK6>Dilp2–FAP boutons co-expressing Rugose RNAi (Rg RNAi) with and without FSK (−FSK: *n*=13 NMJs, 159 boutons, six animals; +FSK: *n*=11 NMJs, 148 boutons, five animals). No significant difference was found between kiss-and-run fusions frequency in FSK-treated and non-treated boutons (unpaired two-tailed *t*-test). 1/min, min^−1^.

In contrast, presynaptic PKA containing the type 2 regulatory subunit (PKA-R2) is required for cAMP-evoked DCV full fusions. First, the PKA catalytic subunit inhibitor H89 (20 µM) blocks the forskolin-induced full fusions (Fig. 5A, white bars) without altering kiss-and-run events (Fig. 5A, black bars). Second, presynaptic expression of a PKA-R1 dominant-negative subunit failed to alter the cAMP effect (Fig. 5B), consistent with the involvement of PKA-R2. Third, knockdown of PKA-R2 inhibited the cAMP effect. For these experiments, initial attempts to express Dilp2–FAP and a PKA-R2 RNAi with the motor neuron driver OK6-GAL4 did not produce viable progeny. Therefore, PKA-R2 RNAi was expressed only in type Ib boutons with the Dip-β-GAL4 driver (Wang et al., 2022). Strikingly, the forskolin effect on emptying frequency was eliminated, whereas kiss-and-run events were unchanged (Fig. 5C,D). We also tested the role of PKA-R2 in type Is boutons using the ShakB-GAL4 driver. Forskolin produced the same phenomenon of increased emptying frequency in type Is boutons as found in Ib boutons (Fig. 5E), and this effect was also inhibited by PKA-R2 knockdown, again without affecting kiss-and-run events (Fig. 5F). Finally, we focused on the association of PKA-R2 with A-kinase-anchoring proteins (AKAPs). Acute treatment of NMJs with 20 µM St-Ht31, a stearated membrane-permeant inhibitor of the interaction of PKA-R2 with AKAPs (Vijayaraghavan et al., 1997), abolished the facilitating effect of FSK on DCV full fusions with no effect on kiss-and-run frequency (Fig. 5G). This short-term experiment further implicates PKA-R2 without confounding developmental effects. However, this result cannot localize the interaction of PKA-R2 and AKAP to the presynaptic neuron (as opposed to the postsynaptic muscle). Therefore, RNAi knockdown of *Drosophila* Rugose (also called Akap550), a neuronal PKA-R2 anchor (Han et al., 1997), was induced in motor neurons. This genetic presynaptic perturbation inhibited the effect of cAMP on DCV full fusions, again without affecting kiss-and-run exocytosis frequency (Fig. 5H). Taken together, these experimental results demonstrate that cAMP-mediated activation of anchored PKA-R2 triggers Ca^2+^-independent full fusion of presynaptic DCVs.

### Complexin participates in cAMP-evoked release from synaptic DCVs

The above results pose the question of how Rugose-anchored PKA activity induces Ca^2+^-independent synaptic DCV full fusions. Recent findings directed our attention to Complexin (Cpx), which is often referred to as a fusion clamp because it reduces spontaneous SSV fusion. First, spontaneous release from DCVs and SSVs are similar at the NMJ: both persist in the presence of tetanus toxin and share SNARE dependence (Bulgari et al., 2019). Furthermore, *Drosophila* Complexin is phosphorylated by PKA at S126 to upregulate (or unclamp) spontaneous SSV release events (Cho et al., 2015). Therefore, the increase in spontaneous DCV release events with cAMP might involve Complexin phosphorylation. Second, *in vitro* reconstitution experiments devoid of much of the cellular exocytosis machinery suggest the potential for facilitation of fusion pore dilation by Complexin (Pierson and Shin, 2021). Together, these observations support the hypothesis that Complexin S126 is required for Ca^2+^-independent PKA-induced spontaneous DCV full fusions.

**Fig. 6.**
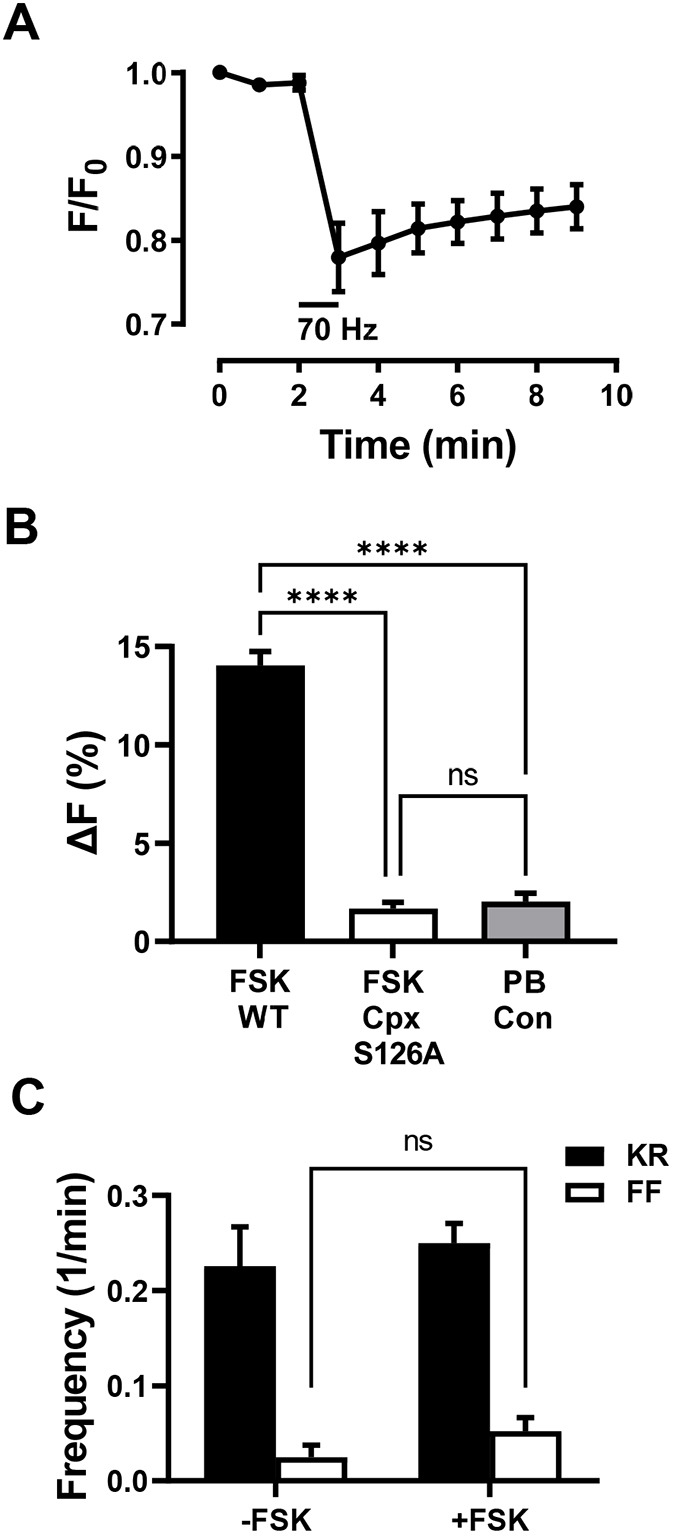
**Complexin participates in cAMP-evoked full fusions and neuropeptide release.** (A) Release indicated by loss of ANF–GFP fluorescence (F) is induced by a 1 min 70 Hz stimulation (indicated by the horizontal bar) in elav-GAL4 UAS-ANF-GFP;;UAS-Cpx^S126A^, Cpx^SH1^ animals. *n*=4 NMJs (one bouton each per animal). (B) Forskolin-evoked ANF–GFP release in 7 min is abolished in elav-GAL4 UAS-ANF-GFP;;UAS-Cpx^S126A^, Cpx^SH1^ animals. WT, wild type Cpx; PB Con, photobleaching control. *n*=4–6 NMJs (1 bouton each per animal). ****P<0.0001; ns, not significant (one-way ANOVA followed by Tukey's test). (C) Frequency of kiss-and-run (KR, black columns) and full fusions (FF, white columns) in boutons from elav-GAL4;UAS-Dilp2-FAP;UAS-Cpx^S126A^, Cpx^SH1^/Cpx^Delta2^ flies in the absence and presence of forskolin (−FSK: *n*=7 NMJs, 53 boutons, four animals; +FSK: *n*=14 NMJs, 124 boutons, six animals). Cpx^SH1^ and Cpx^Delta2^ are null mutants. Note that forskolin does not have a statistically significant effect (ns, not significant by unpaired two-tailed *t*-test) on full fusion frequency (open bars). Likewise, no effect was produced on kiss-and-run frequency. 1/min, min^−1^.

Therefore, we examined the effect of replacing native complexin with the unphosphorylatable S126A mutant (Cpx^S126A^) by rescuing a Cpx-null mutant with neuronal expression of Cpx^S126A^ (i.e. as in Cho et al., 2015). First, to test whether the mutant rescue was effective for DCVs, a GFP-tagged neuropeptide (ANF–GFP) (Rao et al., 2001) that reports native DCV-mediated release (e.g. Husain and Ewer, 2004) was expressed while replacing Cpx with Cpx^S126A^, and release was measured as the loss of ANF–GFP fluorescence. In the presence of extracellular Ca^2+^, activity-evoked DCV-mediated release in Cpx^S126A^ animals was intact – ∼20% release was followed by some recovery due to activity-dependent capture (Fig. 6A). These results match prior control responses with GFP imaging (Shakiryanova et al., 2005; 2006; Wong et al., 2015; Cavolo et al., 2016). Thus, the Cpx^S126A^ mutant supports Ca^2+^-dependent synaptic DCV exocytosis.

Then cAMP-evoked release was measured in the absence of extracellular Ca^2+^. Fig. 6B shows that the ANF–GFP release response to forskolin is abolished in Cpx^S126A^ animals: with the mutant the minor change in fluorescence that occurred with forskolin matched the photobleaching control. Finally, FAP imaging was used to measure the impact of Cpx^S126A^ on DCV full fusions (Fig. 6C). First, basal data are comparable to the results throughout this study. Second, in accordance with ANF–GFP experiments, cAMP stimulation of DCV full fusions was abolished (i.e. the white bars in Fig. 6C were not significantly different), while kiss-and-run events were unaffected. Therefore, the PKA phosphorylation site in Complexin is needed for the cAMP-induced increase in the frequency of spontaneous DCV-mediated full fusions. By evoking the opening of dilating fusion pores, proteins are released at the synapse that are too large to permeate through the fusion pores that normally dominate in the absence of cAMP and Complexin signaling.

## DISCUSSION

Neuronal DCVs are distinguished by their small size relative to the best-studied endocrine DCVs, as well as their widely varying contents within a single cell and, at a native intact synapse, the nearly exclusive use of fusion pores formed by dynamin-dependent kiss-and-run exocytosis for spontaneous and activity-evoked release (Wong et al., 2015; Bulgari et al., 2019; Fig. 3A). The resultant partial release from DCVs (Wong et al., 2015) fits with the need to preserve presynaptic DCVs, which can only be replaced very slowly by axonal transport, but posed the question of whether large DCV cargoes can permeate through fusion pores. FAP imaging with a series of PEGylated fluorogens provided a means to determine the conduction properties of synaptic DCV fusion pores without cargo-specific confounds that affect release (e.g. binding to luminal DCV constituents). The experimental data presented here establish that presynaptic DCV fusion pores are larger than those found in mammalian chromaffin cells (Albillos et al., 1997; Barg et al., 2002) to accommodate the size range of *Drosophila* neuropeptides (0.6–30 kDa), but insufficient for large proteins that undergo activity-dependent synaptic release (e.g. the 70 kDa protease tissue plasminogen activator). However, this limit is bypassed by cAMP-induced Ca^2+^-independent full fusions. At this point, the physiological impact of markedly increasing the release of large proteins over their low basal rates remains to be established. However, we suggest that the cAMP-evoked efficient release of large cargoes might contribute to the development of NMJ terminals, which like DCV full fusions depends on octopamine, cAMP, PKA-R2, PKA-mediated Complexin phosphorylation and neuronal Rugose (Zhong et al., 1992; Koon et al., 2011; Volders et al., 2012; Cho et al., 2015; Vonhoff and Keshishian, 2017). In this context, it is interesting that repeated kiss-and-run events (Wong et al., 2015) might produce synaptic DCVs that have been emptied of neuropeptides, but are still full of large proteins that are poised to affect development and plasticity.

Rugose affects a variety of adult behaviors including associative learning (Volders et al., 2012; Wise et al., 2015). Because Rugose and its mammalian ortholog neurobeachin, a gene implicated in autism (Castermans et al., 2003), are localized near the trans-Golgi network (Wang et al., 2000; Volders et al., 2012), effects on synapses and behavior have been proposed to originate from perturbing the somatodendritic compartment (e.g. by affecting Golgi function, postsynaptic receptors and dendritic spines) (Niemann et al., 2011; Volders et al., 2012; Gromova et al., 2018; Repetto et al., 2018). However, the effect of acute disruption of PKA-R2 anchoring on cAMP-induced full fusions at boutons (Fig. 5G) establishes that Rugose is present and functions in the presynapse. Thus, locally controlled cAMP-induced spontaneous synaptic release by DCVs involving Rugose/neurobeachin-anchored PKA-R2 might influence behavior.

One way the regulation of fusion could be represented is with a channel gating model in which the fusion pore moves between closed (C), open (O) and dilated (D) states (C ⇌ O→D). In this scheme, reaching only the O state results in kiss-and-run release, whereas reaching the D state produces full fusion (i.e. DCV emptying). If the cAMP-PKA-Complexin pathway selectively increased the number of fusions by promoting the C→O step, the frequency of kiss-and-run events, as well as subsequent emptying events, would have increased. However, kiss-and-run frequency did not increase in response to cAMP, thus excluding this possibility. An alternative hypothesis is that cAMP-PKA-Complexin pathway increases the incidence of dilation, which initiates full fusion (the O→D step). With this mechanism, would-be kiss-and-run release events are converted into full fusion events. Therefore, the greater frequency of full fusions would be accompanied with a reduced frequency of kiss-and-run events. But this was not found, thereby showing cAMP-induced full fusions are not produced at the expense of kiss-and-run events. Instead, Rugose-anchored PKA increases total fusion pore openings with the ‘extra’ events being committed to fusion pore dilation. With simple gating models eliminated, the selective increase in spontaneous full fusions reported here is consistent with Complexin phosphorylation both unclamping DCVs normally held in reserve (thus recruiting ‘extra’ DCVs to undergo Ca^2+^-independent exocytosis) and, as proposed from *in vitro* reconstitution experiments (Pierson and Shin, 2021), dilating resultant fusion pores. With pore dilation, large cargoes, such as proteases implicated in synaptic development, which do not readily pass through activity-evoked fusion pores, are released. Thus, diverse exocytosis triggers (Ca^2+^ elevation induced by activity and Rugose-anchored PKA) control fusion pore dilation to differentially gate the release of neuropeptides and large DCV cargoes.

## MATERIALS AND METHODS

### Imaging

*Drosophila melanogaster* third-instar larvae of both sexes were filleted and imaged in Ca^2+^-free HL3 in which 1.5 mM Ca^2+^ was replaced with 0.5 mM EGTA, a Ca^2+^ chelator (70 mM NaCl, 5 mM KCl, 0.5 mM Na_3_EGTA, 20 mM MgCl_2_, 10 mM NaHCO_3_, 5 mM trehalose, 115 mM sucrose, 5 mM hemi-sodium HEPES, pH 7.25). For electrical stimulation experiments they were transferred to HL3 saline that contained (in mM): 70 mM NaCl, 5 KCl, 1.5 CaCl_2_, 20 MgCl_2_, 10 NaHCO_3_, 5 trehalose, 115 sucrose and 5 hemi-sodium HEPES, pH 7.25 supplemented with 10 mM L-glutamate to prevent muscle contractions. Nerve terminals were stimulated at 70 Hz for 1 min via segmental nerves with a suction electrode to evoke maximal release (Shakiryanova et al., 2005). Dilp2–FAP data were acquired in muscle 6 and 7 type I boutons of with an upright Olympus microscope equipped with a 60×1.1 NA water immersion objective, a Yokogawa CSU-X1 spinning disk confocal head, a Coherent Obis 640 nm laser for fluorogen illumination, a Ludl filter wheel with an ET 700/75 emission filter and a Teledyne Photometrics Prime 95B sCMOS camera. All data were derived from type Ib boutons except in Fig. 5E,F in which expression was driven in type Is boutons. ANF–GFP data were acquired as described previously (Levitan et al., 2007; Wong et al., 2015). PACα photoactivation in synaptic boutons was achieved by illuminating synaptic boutons with 488 nm light for 300 ms at 0.33 Hz for the whole duration of FAP imaging (4 min). Quantification of fluorescence intensity was performed with ImageJ software (https://imagej.nih.gov/ij/) as previously described (Levitan et al., 2007). Spontaneous fusion events were counted manually from time-lapse images taken for 4 min at 0.33 or 0.5 Hz. Statistical analysis and graphing were performed with GraphPad Prism software. Error bars represent s.e.m.. Statistical significance was determined with unpaired two-tailed Student's *t*-test for two experimental groups and one-way ANOVA with Dunnett's or Tukey's post-test for more experimental groups.

### Reagents

For synthesis of MG-PEGs, commercially available mPEG-azides of sizes 1 K, 2 K, 5 K, 10 K and 20 K (Biochempeg Scientific Inc.) were coupled to MG-EDA-alkyne via click reaction (Szent-Gyorgyi et al., 2022). Hydrodynamic sizes in terms of apparent protein molecular masses were determined by FPLC with a HiPrep 26/60 Sephacryl S-200 high-resolution column with protein standards ranging from 6.5 to 66.5 kDa (aprotinin, cytochrome C, carbonic anhydrase, albumin). Resultant apparent protein molecular masses were: 4.3 kDa for MG–PEG1K, 12.5 kDa for MG–PEG2K, 32.4 kDa for MG–PEG5K, 53.2 kDa for MG–PEG10K and 71.1 kDa for MG–PEG20K. MG–PEG conjugates and MG–BTau (Bulgari et al., 2019) were used at 1 µM final concentration in imaging experiments. Octopamine and MG–BTau were bath applied immediately before imaging. Time-lapse images were taken for 4 min at 0.3 or 0.5 Hz. Boutons were treated with 8-parachlorophenyl-thio-cAMP and 8-(4-methoxyphenylthio)-2′-O-methyl-cAMP for 15 min before application of 1 µM MG–BTau followed by image sequence acquisition. stHT-31, ESI-09 and H-89 were applied for 15 to 20 min before application of forskolin, then imaged 3 min after forskolin exposure in the presence of MG–BTau. Forskolin was dissolved in DMSO as stock and subsequently diluted to yield a final concentration of DMSO of 0.1%. An equivalent amount of the vehicle was used as the matched control. Forskolin, 8-parachlorophenyl-thio-cAMP and ESI-09 were from Sigma, 8-(4-methoxyphenylthio)-2′-O-methyl-cAMP from Biolog Life Sciences, H-89 from Cayman Chemicals and St-Ht31 from Thermo Fisher Scientific. HRP labeling was undertaken with Alexa Fluor 488-conjugated goat anti-horse radish peroxidase from Jackson Immunoresearch (Code: 123-545-021, Lot: 134496) diluted at 1:100.

### Flies

UAS-Dilp2-FAP and UAS-ANF-GFP flies were described previously (Rao et al., 2001; Bulgari et al., 2019). Epac function was disrupted based a P-element insertion in the gene (Epac KG00434; Bloomington *Drosophila* Stock Center (BDSC) stock #13663). Other BDSC stocks used were: OK6-GAL4 (#64199), DIPβ-GAL4 (#90316), UAS-PACα (#78790), the dominant negative PKA-R1 UAS-PKA.R1BDK (#35550), UAS-PKA-R2 RNAi (#53930), Valium20 RNAi control (#35783), UAS-rugose RNAi (#57703) and the complexin-null Cpx^Delta2^ (#64252). The ShakB-GAL4 line was from Tanja Godenschwege (Florida Atlantic University, Boca Raton, FL, USA). J. Troy Littleton (Massachusetts Institute of Technology, Cambridge, MA, USA) provided elav-GAL4;;UAS-Cpx^S126A^, Cpx^SH1^/TM6B,Tb flies, with Cpx^SH1^ being a Complexin-null mutant.

## References

[JCS261026C1] Albillos, A., Dernick, G., Horstmann, H., Almers, W., Alvarez De Toledo, G. and Lindau, M. (1997). The exocytotic event in chromaffin cells revealed by patch amperometry. *Nature* 389, 509-512. 10.1038/39081 9333242

[JCS261026C2] Anantharam, A., Bittner, M. A., Aikman, R. L., Stuenkel, E. L., Schmid, S. L., Axelrod, D. and Holz, R. W. (2011). A new role for the dynamin GTPase in the regulation of fusion pore expansion. *Mol. Biol. Cell* 22, 1907-1918. 10.1091/mbc.e11-02-0101 21460182 PMC3103406

[JCS261026C3] Baranes, D., Lederfein, D., Huang, Y. Y., Chen, M., Bailey, C. H. and Kandel, E. R. (1998). Tissue plasminogen activator contributes to the late phase of LTP and to synaptic growth in the hippocampal mossy fiber pathway. *Neuron* 21, 813-825. 10.1016/S0896-6273(00)80597-8 9808467

[JCS261026C4] Barg, S., Olofsson, C. S., Schriever-Abeln, J., Wendt, A., Gebre-Medhin, S., Renström, E. and Rorsman, P. (2002). Delay between fusion pore opening and peptide release from large dense-core vesicles in neuroendocrine cells. *Neuron* 33, 287-299. 10.1016/S0896-6273(02)00563-9 11804575

[JCS261026C5] Bulgari, D., Jha, A., Deitcher, D. L. and Levitan, E. S. (2018). Myopic (HD-PTP, PTPN23) selectively regulates synaptic neuropeptide release. *Proc. Natl. Acad. Sci. USA.* 115, 1617-1622. 10.1073/pnas.1716801115 29378961 PMC5816177

[JCS261026C6] Bulgari, D., Deitcher, D. L., Schmidt, B. F., Carpenter, M. A., Szent-Gyorgyi, C., Bruchez, M. P. and Levitan, E. S. (2019). Activity-evoked and spontaneous opening of synaptic fusion pores. *Proc. Natl. Acad. Sci. U.S.A* 116, 17039-17044. 10.1073/pnas.1905322116 31383765 PMC6708360

[JCS261026C7] Calejo, A. I., Jorgaä Evski, J., Rituper, B., Guček, A., Pereira, P. M., Santos, M. A. S., Potokar, M., Vardjan, N., Kreft, M., Gonçalves, P. P. et al. (2014). Hyperpolarization-activated cyclic nucleotide-gated channels and cAMP-dependent modulation of exocytosis in cultured rat lactotrophs. *J. Neurosci* 34, 15638-15647. 10.1523/JNEUROSCI.5290-13.2014 25411492 PMC6608442

[JCS261026C8] Calejo, A. I., Jorgacevski, J., Kucka, M., Kreft, M., Gonçalves, P. P., Stojilkovic, S. S. and Zorec, R. (2013). cAMP-mediated stabilization of fusion pores in cultured rat pituitary lactotrophs. *J. Neurosci* 33, 8068-8078. 10.1523/JNEUROSCI.5351-12.2013 23637196 PMC3674111

[JCS261026C9] Castermans, D., Wilquet, V., Parthoens, E., Huysmans, C., Steyaert, J., Swinnen, L., Fryns, J.-P., Van de Ven, W. and Devriendt, K. (2003). The neurobeachin gene is disrupted by a translocation in a patient with idiopathic autism. *J. Med. Genet.* 40, 352-356. 10.1136/jmg.40.5.352 12746398 PMC1735479

[JCS261026C10] Cavolo, S. L., Bulgari, D., Deitcher, D. L. and Levitan, E. S. (2016). Activity Induces Fmr1-Sensitive Synaptic Capture of Anterograde Circulating Neuropeptide Vesicles. *J. Neurosci.* 36, 11781-11787. 10.1523/JNEUROSCI.2212-16.2016 27852784 PMC5125230

[JCS261026C11] Cho, R. W., Buhl, L. K., Volfson, D., Tran, A., Li, F., Akbergenova, Y. and Littleton, J. T. (2015). Phosphorylation of complexin by PKA regulates activity-dependent spontaneous neurotransmitter release and structural synaptic plasticity. *Neuron* 88, 749-761. 10.1016/j.neuron.2015.10.011 26590346 PMC4847943

[JCS261026C12] de Rooij, J., Zwartkruis, F. J. T., Verheijen, M. H. G., Cool, R. H., Nijman, S. M. B., Wittinghofer, A. and Bos, J. L. (1998). Epac is a Rap1 guanine-nucleotide-exchange factor directly activated by cyclic AMP. *Nature* 396, 474-477. 10.1038/24884 9853756

[JCS261026C13] González-Santana, A., Estévez-Herrera, J., Seward, E. P., Borges, R. and Machado, J. D. (2021). Glucagon-like peptide-1 receptor controls exocytosis in chromaffin cells by increasing full-fusion events. *Cell Rep.* 36, 109609. 10.1016/j.celrep.2021.109609 34433018

[JCS261026C14] Gromova, K. V., Muhia, M., Rothammer, N., Gee, C. E., Thies, E., Schaefer, I., Kress, S., Kilimann, M. W., Shevchuk, O., Oertner, T. G. et al. (2018). Neurobeachin and the kinesin KIF21B are critical of endocytic recycling of NMDA receptor and regulate social behavior. *Cell Rep.* 23, 2705-2717. 10.1016/j.celrep.2018.04.112 29847800

[JCS261026C15] Guček, A., Gandasi, N. R., Omar-Hmeadi, M., Bakke, M., Døskeland, S. O., Tengholm, A. and Barg, S. (2019). Fusion pore regulation by cAMP/Epac2 controls cargo release during insulin exocytosis. *Elife* 8, e41711. 10.7554/eLife.41711 31099751 PMC6557626

[JCS261026C16] Han, J. D., Baker, N. E. and Rubin, C. S. (1997). Molecular characterization of a novel A kinase anchor protein from *Drosophila melanogaster*. *J Biol. Chem.* 272, 26611-26619. 10.1074/jbc.272.42.26611 9334242

[JCS261026C17] Huang, Y. Y., Bach, M. E., Lipp, H. P., Zhuo, M., Wolfer, D. P., Hawkins, R. D., Schoonjans, L., Kandel, E. R., Godfraind, J. M., Mulligan, R. et al. (1996). Mice lacking the gene encoding tissue-type plasminogen activator show a selective interference with late-phase long-term potentiation in both Schaffer collateral and mossy fiber pathways. *Proc. Natl. Acad. Sci. USA* 93, 8699-8704. 10.1073/pnas.93.16.8699 8710934 PMC38736

[JCS261026C18] Husain, Q. M. and Ewer, J. (2004). Use of targetable gfp-tagged neuropeptide for visualizing neuropeptide release following execution of a behavior. *J. Neurobiol.* 59, 181-191. 10.1002/neu.10309 15085536

[JCS261026C19] Klose, M. K., Bruchez, M. P., Deitcher, D. L. and Levitan, E. S. (2021). Temporally and spatially partitioned neuropeptide release from individual clock neurons. *Proc. Natl. Acad. Sci. U.S.A* 118, e2101818118. 10.1073/pnas.2101818118 33875606 PMC8092580

[JCS261026C20] Koon, A. C., Ashley, J., Barria, R., Dasguta, S., Brain, R., Waddell, S., Alkema, M. J. and Budnik, V. (2011). Autoregulatory and paracrine control of synaptic and behavioral plasticity by octopaminergic signaling. *Nat. Neurosci.* 14, 190-199. 10.1038/nn.2716 21186359 PMC3391700

[JCS261026C21] Kucka, M., Bjelobaba, I., Tomić, M. and Stojilkovic, S. S. (2013). The role of cyclic nucleotides in pituitary lactotroph functions. *Front. Endocrinol. (Lausanne)* 4, 122. 10.3389/fendo.2013.00122 24062725 PMC3772395

[JCS261026C22] Levitan, E. S., Lanni, F. and Shakiryanova, D. (2007). *In vivo* imaging of vesicle motion and release at the *Drosophila* neuromuscular junction. *Nat. Protoc.* 2, 1117-1125. 10.1038/nprot.2007.142 17546002

[JCS261026C23] Maiellaro, I., Lohse, M. J., Kittel, R. J. and Calebiro, D. (2016). cAMP Signals in *Drosophila* motor neurons are confined to single synaptic boutons. *Cell Rep.* 17, 1238-1246. 10.1016/j.celrep.2016.09.090 27783939 PMC5098120

[JCS261026C24] Niemann, K., Breuer, D., Brockhaus, J., Born, G., Wolff, I., Reissner, C., Kilimann, M. W., Rohlmann, A. and Missler, M. (2011). Dendritic spine formation and synaptic function require Neurobeachin. *Nat. Comm.* 2, 557. 10.1038/ncomms1565 PMC3482631 22109531

[JCS261026C25] Perkins, L. A., Yan, Q., Schmidt, B. F., Kolodieznyi, D., Saurabh, S., Larsen, M. B., Watkins, S. C., Kremer, L. and Bruchez, M. P. (2018). Genetically targeted ratiometric and activated pH indicator complexes (TRApHIC) for receptor trafficking. *Biochemistry* 57, 861-871. 10.1021/acs.biochem.7b01135 29283245

[JCS261026C26] Pierson, J. and Shin, Y. K. (2021). Stabilization of the SNARE core by Complexin-1 facilitates fusion pore expansion. *Front. Mol. Biosci.* 8, 805000. 10.3389/fmolb.2021.805000 34970598 PMC8712692

[JCS261026C27] Rao, S., Lang, C., Levitan, E. S. and Deitcher, D. L. (2001). Visualization of neuropeptide expression, transport, and exocytosis in *Drosophila melanogaster*. *J. Neurobiol* 49, 159-172. 10.1002/neu.1072 11745655

[JCS261026C28] Repetto, D., Brockhaus, J., Rhee, H. J., Lee, C., Kilimann, M. W., Rhee, J., Northoff, L. M., Guo, W., Reissner, C. and Missler, M. (2018). Molecular dissection of Neurobeachin function at excitatory synapses. *Front. Synaptic. Neurosci.* 10, 28. 10.3389/fnsyn.2018.00028 30158865 PMC6104133

[JCS261026C29] Schröder-Lang, S., Schwärzel, M., Seifert, R., Strünker, T., Kateriya, S., Looser, J., Watanabe, M., Kaupp, U. B., Hegemann, P. and Nagel, G. (2007). Fast manipulation of cellular cAMP level by light in vivo. *Nat. Methods* 4, 39-42. 10.1038/nmeth975 17128267

[JCS261026C30] Shakiryanova, D., Tully, A., Hewes, R. S., Deitcher, D. L. and Levitan, E. S. (2005). Activity-dependent liberation of synaptic neuropeptide vesicles. *Nat. Neurosci.* 8, 173-178. 10.1038/nn1377 15643430

[JCS261026C31] Shakiryanova, D., Tully, A. and Levitan, E. S. (2006). Activity-dependent synaptic capture of transiting peptidergic vesicles. *Nat. Neurosci.* 9, 896-9000. 10.1038/nn1719 16767091

[JCS261026C32] Shakiryanova, D., Zettel, G. M., Gu, T., Hewes, R. S. and Levitan, E. S. (2011). Synaptic neuropeptide release induced by octopamine without Ca^2+^ entry into the nerve terminal. *Proc. Natl. Acad. Sci. USA* 108, 4477-4481. 10.1073/pnas.1017837108 21368121 PMC3060249

[JCS261026C33] Shafer, O. T., Kim, D. J., Dunbar-Yaffe, R., Nikolaev, V. O., Lohse, M. J. and Taghert, P. H. (2008). Widespread receptivity to neuropeptide PDF throughout the neuronal circadian clock network of *Drosophila* revealed by real-time cyclic AMP imaging. *Neuron* 58, 223-237. 10.1016/j.neuron.2008.02.018 18439407 PMC2586874

[JCS261026C34] Sharma, S. and Lindau, M. (2018). The fusion pore, 60 years after the first cartoon. *FEBS Lett.* 592, 3542-3562. 10.1002/1873-3468.13160 29904915 PMC6231997

[JCS261026C35] Stenovec, M., Kreft, M., Poberaj, I., Betz, W. J. and Zorec, R. (2004). Slow spontaneous secretion from single large dense-core vesicles monitored in neuroendocrine cells. *FASEB J.* 18, 1270-1272. 10.1096/fj.03-1397fje 15180959

[JCS261026C36] Sturman, D. A., Shakiryanova, D., Hewes, R. S., Deitcher, D. L. and Levitan, E. S. (2006). Nearly neutral secretory vesicles in *Drosophila* nerve terminals. *Biophys. J.* 90, L45-L47. 10.1529/biophysj.106.080978 16428282 PMC1386803

[JCS261026C37] Szent-Gyorgyi, C., Perkins, L. A., Schmidt, B. F., Liu, Z., Bruchez, M. P. and Van De Weerd, R. (2022). Bottom-Up design: a modular golden gate assembly platform of yeast plasmids for simultaneous secretion and surface display of distinct FAP fusion proteins. *ACS Synth. Biol.* 11, 3681-3698. 10.1021/acssynbio.2c00283 36260923

[JCS261026C38] Takahashi, N., Kishimoto, T., Nemoto, T., Kadowaki, T. and Kasai, H. (2002). Fusion pore dynamics and insulin granule exocytosis in the pancreatic islet. *Science* 297, 1349-1352. 10.1126/science.1073806 12193788

[JCS261026C39] Vijayaraghavan, S., Goueli, S. A., Davey, M. P. and Carr, D. W. (1997). Protein kinase A-anchoring inhibitor peptides arrest mammalian sperm motility. *J. Biol. Chem.* 272, 4747-4752. 10.1074/jbc.272.8.4747 9030527

[JCS261026C40] Volders, K., Scholz, S., Slabbaert, J. R., Nagel, A. C., Verstreken, P., Creemers, J. W. M., Callaerts, P. and Schwarzel, M. (2012). *Drosophila* rugose is a functional homolog of mammalian Neurobeachin and affects synaptic architecture, brain morphology, and associative learning. *J. Neurosci.* 32, 15193-15204. 10.1523/JNEUROSCI.6424-11.2012 23100440 PMC6704825

[JCS261026C41] Vonhoff, F. and Keshishian, H. (2017). Cyclic nucleotide signaling is required during synaptic refinement at the *Drosophila* neuromuscular junction. *Dev. Neurobiol.* 77, 39-60. 10.1002/dneu.22407 27281494 PMC5148738

[JCS261026C42] Wang, X., Herber, F. W., Laue, M. M., Wullner, C., Hu, B., Petrasch-Parwez, E. and Kilimann, M. W. (2000). Neurobeachin: A protein kinase A-anchoring, beige/Chediak-higashi protein homolog implicated in neuronal membrane traffic. *J. Neurosci.* 20, 8551-8565. 10.1523/JNEUROSCI.20-23-08551.2000 PMC6773050 11102458

[JCS261026C43] Wang, Y., Lobb-Rabe, M., Ashley, J., Chatterjee, P., Anand, V., Bellen, H. J., Kanca, O. and Carrillo, R. A. (2022). Systematic expression profiling of Dpr and DIP genes reveals cell surface codes in *Drosophila* larval motor and sensory neurons. *Development* 49, dev200355. 10.1242/dev.200355 PMC9188756 35502740

[JCS261026C44] Wise, A., Tenezaca, L., Fernandez, R. W., Schatoff, E., Flores, J., Ueda, A., Zhong, X., Wu, C.-F., Simon, A. F. and Venkatesh, T. (2015). *Drosophila* mutants of the autism candidate gene neurobeachin (rugose) exhibit neuro-developmental disorders, aberrant synaptic properties, altered locomotion, and impaired adult social behavior and activity patterns. *J. Neurogenet.* 29, 135-143. 10.3109/01677063.2015.1064916 26100104 PMC4747641

[JCS261026C45] Wong, M. Y., Cavolo, S. L. and Levitan, E. S. (2015). Synaptic neuropeptide release by dynamin-dependent partial release from circulating vesicles. *Mol. Biol. Cell* 26, 2466-2474. 10.1091/mbc.E15-01-0002 25904335 PMC4571301

[JCS261026C46] Zhong, Y., Budnik, V. and Wu, C. F. (1992). Synaptic plasticity in Drosophila memory and hyperexcitable mutants: role of cAMP cascade. *J. Neurosci.* 12, 644-651. 10.1523/JNEUROSCI.12-02-00644.1992 1371316 PMC6575602

